# Prevalence of polymorphisms in glucose-6-phosphate dehydrogenase, sickle haemoglobin and nitric oxide synthase genes and their relationship with incidence of uncomplicated malaria in Iganga, Uganda

**DOI:** 10.1186/s12936-017-1970-1

**Published:** 2017-08-09

**Authors:** Catherine Nassozi Lwanira, Fred Kironde, Mark Kaddumukasa, Göte Swedberg

**Affiliations:** 1 School of Biomedical Sciences, College of Health Sciences, Makerere University, Kampala, Uganda; 2 Habib Medical School, Faculty of Health Sciences, Islamic University in Uganda (IUIU), Kampala Campus, Kampala, Uganda; 3 School of Medicine, College of Health Sciences, Makerere University, Kampala, Uganda; 4 Department of Medical Biochemistry and Microbiology, Uppsala University, Uppsala, Sweden

**Keywords:** Human gene polymorphisms, *Plasmodium falciparum* malaria, Incidence

## Abstract

**Background:**

Host genetics play an important role in *Plasmodium falciparum* malaria susceptibility. However, information on host genetic factors and their relationships with malaria in the vaccine trial site of Iganga, Uganda is limited. The main objective of this study was to determine the prevalence of selected host genetic markers and their relationship to malaria incidence in the vaccine trial site of Iganga, Uganda. In a 1-year longitudinal cohort study, 423 children aged below 9 years were recruited and their malaria episodes were investigated. Host genetic polymorphisms were assessed by PCR–RFLP, haemoglobin electrophoresis and DNA sequencing. Using a multivariate negative binomial regression model, estimates of the impact of human genetic polymorphisms on malaria incidence were performed. In all statistical tests, a P value of <0.05 was considered as significant.

**Results:**

The prevalences of sickle cell haemoglobin trait, G6PD c.202 G>A (rs 1050828) and NOS2 −954 G>C (rs 1800482) variants were 26.6, 22.7 and 17.3%, respectively. Inducible nitric oxide synthase 2 (NOS2 −954 G>C; rs 1800482) heterozygosity was associated with lower incidence of malaria in all age groups {Adjusted incident rates ratio (aIRR) 0.59; 95% CI [0.386–0.887]; P = 0.012)}. About 4% of study subjects had co-existence of sickle cell Hb trait and G6PD deficiency. Sickle cell Hb heterozygotes (Hb AS) aged less than 1 year experienced significantly more malaria episodes annually than children with normal haemoglobin (Hb AA) {aIRR = 1.98; 95% CI [1.240–3.175]; P = 0.004}. There was no significant influence of the sickle cell trait on malaria incidence among older children of 1–9 years.

**Conclusions:**

Mutation (NOS2 −954 G>C; rs 1800482) of nitric oxide synthase 2 gene promoter was associated with a lower incidence of acute malaria. The normal haemoglobin (wild genotype; HbAA) was associated with reduced malaria incidence rates during the first year of life. More understanding of the interplay between host genetics and malaria susceptibility is required.

## Background

The influence of human genetic factors on progression and severity of *Plasmodium falciparum* malaria has been extensively studied and a number of host genes have been suggested to confer specific protection. However, the sickle cell trait and glucose-6-phosphate dehydrogenase (G6PD) deficiency stand out among other host genetic markers reported in genome wide association and multicentre studies [1–3]. Various studies in African countries have reported that sickle cell trait (HbAS) protects against severe malaria in 70–90% cases [4–6] and prevents up to 75% of hospital admissions due to malaria [7]. In other studies, HbAS has been associated with reduced malaria incidence rates [6–9] and delayed onset of malaria [10]. More recently, larger association studies have also consistently linked the sickle cell trait with protection against uncomplicated and severe malaria [3, 11].

G6PD deficiency affects over 400 million people globally, 15-30% of whom are found in sub Saharan Africa [12, 13]. More than 160 G6PD genetic variants have been identified including *G6PD* B (wild type), *G6PD* A (non-deficient type) and *G6PD* A^−^ (African deficient type); however the most common and more frequently associated with G6PD deficiency in sub Saharan Africa is the 376G/202A haplotype [13]. While the protective effect of the HbAS against malaria seems to be clearer, associations with G6PD deficiency vary widely in the different studies, with protection observed in females [14, 15], in males [16], in both [17] and no protection at all [18, 19]. More recently, increasing levels of G6PD deficiency were shown to protect against cerebral malaria, but increasing the risk of severe malaria anaemia in both males and females in multicentre investigations [3, 13]. Notably, many of the earlier studies were largely case–control and examined effects of G6PD deficiency on the risk of severe malaria but not the incidence of uncomplicated malaria. Also, these studies were done in other populations outside Uganda. Only two longitudinal studies that were done in Uganda [20] and Gabon [21] found an increase in the malaria incidence rates among female heterozygotes.

Other host polymorphisms within the inducible nitric oxide synthase 2 (*NOS2*) gene promoter also appear to be protective against severe malaria [22] and uncomplicated malaria [20]. Particularly, a single nucleotide polymorphism (N*OS2* −954 G>C; rs 1800482) is believed to modify NOS2 transcription and increases nitric oxide activity [22]. In previous studies, increased levels of nitric oxide were shown to be important in parasite clearance [22, 23] and protection against *P. falciparum* infection [24–26], yet few studies have investigated the relationship between NOS2 polymorphisms and the incidence of uncomplicated malaria. One particular study from Uganda associated the N*OS2* −954 G>C, but not the −1173 C>T promoter polymorphism (rs 9282799) with reduced malaria incidence rates [20]. In other studies, NOS2 −954 G>C polymorphism heterozygosity was associated with protection against severe malaria [22], yet else where, no relation between *NOS2* polymorphisms and susceptibility to malaria [27] or asymptomatic malaria [28] was observed.

In the present study, the associations between the incidence of uncomplicated malaria and *HBB*, *G6PD* and *NOS2* gene polymorphisms were determined in a 1 year follow up study. Understanding the prevalence of these genetic factors and their impact on malaria incidence is important in providing baseline data of value in malaria vaccine trials and other malaria control interventions.

## Methods

### Participant recruitment

A baseline survey was carried out and eligible children were enrolled into a cohort which was followed up for 1 year. At the study site in Iganga, malaria transmission largely occurs throughout the year [29, 30], but with minor transmission peaks being observed following the major rains which usually occur from April to June and from September to December. In this project, the study children were recruited during November and followed up for 1 year. The inclusion criteria, detailed enrolment and follow up are described in an earlier publication [31]. Study personnel sought for verbal community consent to participate in a brief demographic survey, and written informed consent of each child’s guardian was subsequently obtained before enrolment into the study. Using a standardized questionnaire, demographics and malaria indicator information were collected.

### Active case detection and determination of malaria incidence

After the baseline survey, parents or guardians were instructed to bring their children to the study malaria clinic based at Iganga Hospital whenever the children felt unwell. In addition, study children were actively followed up by two home visits at convenient times of day, on Mondays and Thursdays, every week. A standardized questionnaire was administered for collecting information regarding any illnesses that had occurred since the last visit, use of health care facilities and medications used. At each visit, the tympanic temperature was recorded using a digital thermometer. When fever (tympanic temperature of ≥37.5 °C) or history of recent fever (within the last 24 h) was observed or reported for any study child, a rapid diagnostic test (RDT, OPTIMAL^®^) and microscopy of a stained blood smear were performed to determine the presence of malaria parasites. Malaria was defined as i) the child being ill or having any symptom of malaria illness and ii) the child having fever (tympanic temperature ≥37.5 °C) or a history of fever (within the past 24 h) plus iii) the child having any *P. falciparum* parasitaemia. Sick children found with malaria parasitaemia were administered artemisinin combination therapy (ACT) at the study clinic following Uganda national treatment guidelines [32]. The number of malaria episodes/child over the 1 year of active follow up was documented.

### Microscopy and blood sample collection

Thick blood smears were stained with 2% Giemsa for 30 min. Parasite densities were determined by counting the number of asexual parasites per 200 white blood cells (WBC) and assuming a WBC count of 8000/µL of blood [33]. A smear was judged to be negative if no parasites were seen after examination of one hundred high-power (100× objective) microscope fields. A second microscopist, who was blinded to the results of the first reading, re-read all slides. A third reviewer resolved discordant results. The presence of sexual forms of the parasites was also determined during the slide readings.

In addition, approximately 2 mL of blood were drawn and mixed with ethylene diamine tetracetic acid (EDTA) anticoagulant for subsequent analysis of DNA. Time at risk for new infection was considered as the duration of study participation excluding 14 days after each ACT-treated episode of malaria. Severely malnourished children [34] were not enrolled into the study.

### Genotyping

Genomic DNA was extracted from blood leukocytes using E.Z.N.A Blood DNA kit (Omega Bio-tek, USA) following the manufacturer’s protocol. Assessment of host gene polymorphism was performed by amplifying specific gene fragments with polymerase chain reaction (PCR) followed by endonuclease restriction fragment length polymorphism (RFLP) as described elsewhere [12, 20, 22, 35].

For each reaction, 1–2 μL of the extracted DNA sample was incubated with DreamTaq DNA polymerase (Thermo scientific Inc, USA), 0.1 μM of each primer, 200 μM dNTPs and 2.0 mM MgCl_2_. *HBB* gene amplification was performed using nested PCR in which the products from the first PCR round were subjected to a second amplification using DreamTaq DNA polymerase (Thermo scientific Inc, USA), 0.05 µM of each primer, 200 µM dNTPs and 2.0 mM MgCl_2._ About 5 μL of each amplified product was digested with restriction endonucleases and products were subjected to electrophoresis on 2.5% agarose gels (Thermo scientific Inc, USA) and visualized with ethidium bromide. Genotypes were assessed by comparing the sizes of reaction products and controls after digestion. Mutants were confirmed by DNA sequencing and hemoglobin electrophoresis. DNA sequencing was done using dye terminator chemistry (Applied Biosystems) followed by DNA analysis with ABI3730XL sequencer. Specific oligonucleotide primers that were used for PCR amplification of the host gene segments are shown in Table 1.

**Table 1:** Specific oligonucleotide primers used for PCR amplification

SNP (gene)	PCR round	PCR primers	Product size (bp)	Digesting enzyme	Fragment size (bp)	
					Digested (normal)	Digested (mutant)
rs 334 (*HBB*)	First	5′-AGAAAACATCAAGGGTCCCA-3′ (forward)	926			
		5′-TCCATCTACATATCCCAAAGC-3′ (reverse)				
	Nested	5′-TCCAAGGGTAGACCACCAGC-3′ (forward)	445	*Dde*I	225, 220	445
		5′-GTGCCAGAAGAGCCAAGGAC-3′ (reverse)				
rs 1050828 (*G6PD*)	Single	5′-GTGGCTGTTCCGGGATGGCCTTCTG-3′ (forward)	109	*Nla*II	109	63, 46
		5′-CTTGAAGAAGGGCTCACTCTGTTTG-3′ (reverse)				
rs 1800482 (*NOS2*)	Single	5′-CATATGTATGGGAATACTGTATTTCAGGC-3′	573	*Bsa*I	446, 127	573
		5′-TCTGAACTAGTCACTTGAGG-3′				

### ABO blood group typing

Blood groups of the study children were determined using direct haemagglutination test with monoclonal anti-sera to blood group antigens A, B and RhD [36].

### Data management and analysis

Data were cleaned, coded and entered into Microsoft Office Access 2007. Descriptive statistics, Chi- square tests and multivariate analysis were carried out using Stata12.0 (Stata Corp, College Station, Texas, USA). Allele and genotype frequencies were calculated according to Hardy–Weinberg equation [37]. Associations between host polymorphism and malaria incidence were determined using a multivariate negative binomial regression model that controlled for other independent predictors of malaria risk such as age, malaria history and insecticide treated bed net (ITN) use. Adjusted incidence rate ratios (aIRRs), P values and 95% confidence intervals were calculated. G6PD c.202 G>A male hemizygotes and female homozygotes were coded separately to allow for examination of the effect of each genotype individually. However, the numbers of female homozygotes were too small to yield a confident result; thus another multivariate regression analysis that considered a combination of G6PD c.202 G>A male hemizygotes and female homozygotes was performed. All statistical tests were two -tailed and P values <0.05 were considered significant.

## Results

Cohort children (n = 434) were actively followed up for 1 year and the incidence of malaria (annual episodes per child) was determined. Of the cohort children, 2.5% (11/434) did not provide an adequate blood sample for subsequent analysis of DNA and thus, only 423 children who had been actively followed up were included in the host genetics studies.

### Patient demographic characteristics

Majority of the study participants (96.7%) were of *Basoga* ethnic tribe. Sixty-five percent (274/423) were within the age range of 3–9 years and only 35% (148/423) were aged 0.5–3 years; the mean age was 3.9 years (SD: ±2.3). Slightly over half of the study participants (52.7%) were males. At recruitment, mean hemoglobin was 12 g/dL (SD: ±1.5) {reference range = 8.8–12.5 g/dL} [38] and mean weight was 15.5 kg (SD ± 5.2). The predominant blood groups were O+ (39.4%) and B+ (30.4%).

### Malaria occurrence and indicators

Majority of the guardians of study participants (94.6%) reported that their children had experienced fever during the past 6 months preceding enrolment into the study. At enrolment to the study, approximately 40% (168 of the 423 children) had parasitaemia, with a median parasitaemia of 575 parasites/μL [inter quartile range (IQR) = 225–2750/μL]. About 88.2% of the participants’ guardians reported owning and using an insecticide treated bed net (ITN) within their households, while 95.3% reported having ever administered an anti-malarial drug to the enrolled child. Throughout the 1-year of longitudinal follow up in this study, malaria episodes were not registered among 217 out of 423 children (51.3%). Among those who experienced malaria episodes (206 children; 48.7%) during the 1 year of follow up, the range of annual episodes per child was 1 to 9 (median 4, IQR 3–5).

### Distribution of host genetic variants

Genotyping of G6PD c.202 G>A (rs 1050828), HBB c.20 A>T (rs 334) and NOS2 −954 G>C (rs 1800482) was performed by PCR for all 423 children who were successfully followed up. DNA amplification by PCR was successful in 97% of the analysed samples. The distribution of G6PD genotypes was as follows: 16.3% (69 of 423) were G6PD c.202 G>A heterozygous females; 1% (4 of 423) were homozygous females and 5.4% (23 of 423) were G6PD c.202 G>A hemizygous males. The prevalence of Hb AS was 26.6% and only one child was homozygous for sickle haemoglobin (Hb SS). Interestingly, 3.9% (16 of 414) of the children had both the sickle cell trait and G6PD c.202 G>A variant. The only variant allele at the NOS2 locus that was examined in this study population was NOS2 −954 G>C (rs 1800482) and it was found to occur at a frequency of 9%. The genotype and allele frequencies of G6PD c.202 G>A (rs 1050828), HBB c.20 A>T (rs 334) and NOS2 −954 G>C (rs 1800482) variants are shown in Table 2. The frequencies of HBB, G6PD, and NOS2 polymorphisms of children lacking malaria symptoms throughout the year and those showing malaria symptoms were statistically similar.

**Table 2:** Genotype and allele frequencies of the studied host gene polymorphisms

G6PD c.202 G>A (n = 423)		HBB c.20 A>T (n = 414)		NOS2 −954 G>C (n = 411)	
	Frequency N (%)		Frequency N (%)		Frequency N (%)
Females					
Wild type	158 (37.4)	Wild type, AA	303 (73.2)	Wild type	340 (82.7)
Heterozygous	69 (16.3)	Heterozygous, AS	110 (26.6)	Heterozygous	66 (16.1)
Homozygous	4 (1.0)	Homozygous, SS	1 (0.2)	Homozygous	5 (1.2)
Males					
Wild type	169 (39.9)				
Hemizygous	23 (5.4)				
Allele frequency					
G	0.88	A	0.86	G	0.91
A	0.12	T	0.14	C	0.09

### The relationship between host gene variants and incidence of malaria

During the 1 year of longitudinal follow-up, a total of 414 new episodes of malaria were recorded. As defined above, these episodes comprised of a child being unwell, having any level of parasitaemia and having a fever either at the time of visit to the study clinic or within the previous 24 h. The overall incidence of these episodes was 0.98 per child/year. Peak incidence (1.25 episodes/child/year) occurred between the age range of 1–3 years, which was about 1.8 times the incidence for older children in the age range of 5–9 years (0.70 episodes/child/year). These episodes of ongoing or recent (past 24 h) febrile illness, that were accompanied by *P. falciparum* infection, were included in the final multivariate negative binomial regression model to determine the extent to which they were affected by ABO blood group, HBB c.20 A>T (rs 334), G6PD c.202 G>A (rs 1050828) and NOS2 −954 G>C (rs 1800482) gene variants. None of the ABO bloodgroups showed any significant influence on malaria incidence rates in this cohort. In a previous study in the same cohort of children, age, prior malaria history (reported by guardian) and ITN use were identified as independent predictors of malaria incidence [31].

Overall (see Table 3), there was no dramatic difference between incidence of malaria among sickle cell heterozygotes (Hb AS) and children with normal haemoglobin (HbAA). The adjusted incidence rate ratio (aIRR), was 1.04 (95% CI 0.755–1.427; P = 0.82). With only one child homozygous for *HBB* sickle cell gene, we could not draw any conclusions about effect of sickle cell homozygosity (Hb SS) on malaria incidence.

**Table 3:** Effect of host genetic polymorphism on incidence of malaria

Host polymorphism	Malaria		Number of new episodes	Adjusted incidence rate ratio	P value	95% CI
	No	Yes				
G6PD c.202 G>A						
Wild type	166	161	318	Reference	–	–
Heterozygous	37	32	59	0.98	0.918	0.672–1.429
Homozygous	3	1	3	1.38	0.673	0.313–6.033
Hemizygous	11	12	34	1.60	0.081	0.944–2.698
Homo/hemizygous	14	13	37	1.57	0.076	0.954–2.590
HBB c.20 A>T						
Wild type	158	145	289	Reference	–	–
Heterozygous	57	53	110	1.04	0.818	0.755–1.427
NOS2 −954 G>C						
Wild type	169	171	358	Reference	–	–
Heterozygous	40	26	41	0.59	0.012	0.386–0.887
Homozygous	2	3	5	1.29	0.683	0.383–4.333

Only one child was homozygous for HBB c.20A>T variant and was not included in this analysis

From an identical multivariate negative binomial regression analysis as above, there was no significant difference between the malaria incidence rates for G6PD c.202 G>A heterozygous females and wild-type individuals (aIRR = 0.98; 95% CI [0.755–1.427]; P = 0.92). Only G6PD c.202 G>A hemizygous males showed slightly higher incidence of uncomplicated malaria than the wild-type individuals (aIRR = 1.60; 95% CI [0.944–2.698]; P = 0.08). With only four female homozygotes, it was not possible to obtain a confident conclusion of the effect of G6PD c.202 G>A homozygosity on the incidence of uncomplicated malaria (aIRR = 1.38; 95% CI [0.313–6.033]; P = 0.67). Another multivariate regression analysis was then performed but considering a combination of the female homozygotes and male hemizygotes. G6PD c.202 G>A homo/hemizygous children had slightly higher incidence of uncomplicated malaria reaching 1.37 annual episodes per child compared to 0.97 for G6PD c.202 G>A wild-type (aIRR = 1.57; 95% CI [0.954–2.590]; P = 0.08). Only few children (16 of 416) showed co-existence of sickle cell trait and the G6PD c.202 G>A variant, thus it was not possible to evaluate the effect of G6PD deficiency on sickle trait protection against malaria in this childrens’ cohort.

The only variant that showed significant influence on malaria incidence was the NOS2 −954 G>C (rs 1800482) promoter variant. Crude annual malaria incidence rates were 1.05 episodes per child for the wildtypes and 0.62 per child for heterozygotes. On analysis, NOS2 −954 G>C heterozygotes had significantly lower malaria incidence rates as compared to those without the mutation (aIRR = 0.59; 95% CI [0.386–0.887]; P = 0.012). Only five individuals carried the homozygous mutation and, therefore, the significance of difference between the malaria incidence for homozygotes and wild-type individuals could not be determined accurately (aIRR = 1.29; 95% CI [0.383–4.333]; P = 0.683).

### Effect of age on the relationship between sickle cell trait, G6PD deficiency and malaria incidence

**Fig. 1:**
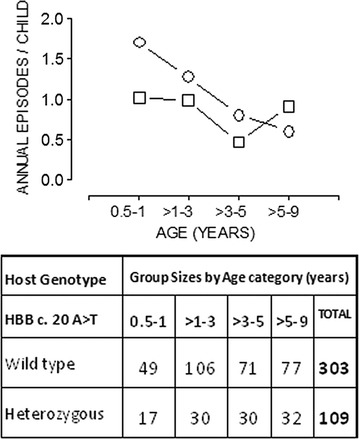
Effect of age on the relationship between sickle cell trait and malaria incidence. Using data from a longitudinal follow up of cohort children, the means (±standard deviation) of annual malaria episodes per child (incidence rates) for sickle cell *HBB* heterozygotes (Hb AS, *cycle symbols*) and wild types (Hb AA, *rectangle symbols*) over four age categories are presented. Children with wild type Hb (AA) experienced lower incidences in early infancy (till age of 1 year) than sickle cell *HBB* heterozygotes. Through ages >1–9 years, malaria incidences were largely similar

An age stratified analysis was carried out, adjusting for all the other predictors of malaria incidence including malaria history as reported by guardian, bloodgroup, ITN use and prior antimalarial drug use. As shown in Fig. 1, sickle cell carriers in the age group of 0.5–1 years showed higher malaria incidence compared to those with normal haemoglobin (Hb AA) [aIRR = 1.984; 95% CI [1.240–3.175]; P = 0.004]. There was no significant difference in malaria incidence experienced by sickle cell Hb heterozygotes and normal Hb individuals among the older age groups of >1–9 years.

**Fig. 2:**
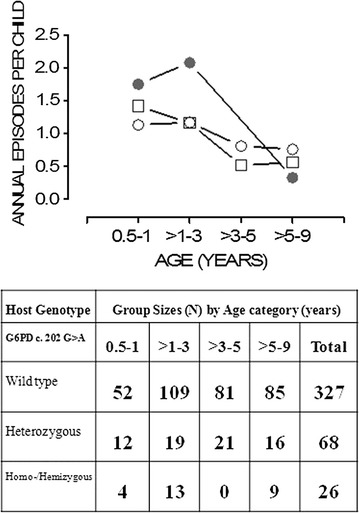
Effect of age on the relationship between G6PD deficiency and malaria incidence. A plot of incidence rates versus age for the different G6PD genotypes is shown. Incidence rates were highest among the G6PD c.202 G>A homozygotes (*closed circles*) and lowest among those without mutations (wildtypes shown by *open circles*). Heterozygous (G6PD c.202 G>A, *open squares*) children experienced malaria incidences less than the homozygous individuals. The adjusted incident rates ratios (aIRR) in reference to G6PD c.202 G>A wild-types for age 6 months to 1 year were; G6PD c.202 G>A homo/hemizygotes (aIRR = 1.54; 95% CI [0.689–3.434]; P = 0.29), G6PD c.202 G>A heterozygotes (aIRR = 1.48; 95% CI [0.838–2.604]; P = 0.18). Peak incidence rates among the homozygotes are seen among the 1–3 year olds {(aIRR = 2.32; 95% CI [1.509–3.559]; P = 0.000), G6PD c.202 G>A heterozygotes (aIRR = 1.01; 95% CI [0.629–1.608]; P = 0.98)}. Older homozygotes (5–9 years) showed the lowest incidence {(aIRR = 0.16; 95% CI [0.021–1.133]; P = 0.07), G6PD c.202 G>A heterozygotes (aIRR = 0.94; 95% CI [0.462–1.921]; P = 0.87). There were no homozygotes in the >3–5 age group

G6PD c.202 G>A homo/hemizygous children older than 1–3 years experienced higher malaria incidence than their wildtype counterparts (aIRR = 2.32; 95% CI [1.509–3.559]; P ≤ 0.001). Observable differences in malaria incidence were not seen among the G6PD c.202 G>A genotypes in the other age categories (Fig. 2).

## Discussion

Susceptibility or resistance to infection by *Plasmodium falciparum* has previously been reported to be influenced by human genetic factors in large genome wide association studies [1, 2] and multicentre investigations [3]. However these studies were largely case–control and examined host genetic effects on the risk of severe malaria in other populations. In the present study, the prevalences of *G6PD c.202 G*>*A (rs 1050828)*, *HBB c.20A*>*T (rs 334)* and *NOS2* −*954 G*>*C (rs 1800482)* gene polymorphisms and their impact on incidence of uncomplicated in the clinical trials site of Iganga in Eastern Uganda was determined. This is an extension of an earlier longitudinal study [31] and benefits from data previously collected regarding the independent predictors of malaria risk in the same children’s cohort [31]. Adjustment for these confounders allowed for determination of the effect of the selected host gene markers on incidence of uncomplicated malaria in the study cohort. The frequencies of the sickle cell trait, G6PD c.202A and NOS2 −954 C were similar to those reported in other African countries [3, 13, 22].

Independent of age, the most significant effect observed in this study was the heterozygous NOS2 −954 G>C mutation, which showed a lower incidence of uncomplicated malaria. This single nucleotide polymorphism has been shown to modify NOS2 transcription and increase nitric oxide activity that may be important in parasite clearance [22, 23] and protection against *P. falciparum* infection [24–26]. An earlier study that was done in Kampala, Uganda showed reduction in the incidence of mild malaria among NOS2 −954 G>C heterozygotes [20]. Also, in the present study, the major ethnic group were the Bantu, a feature that is similar to the Kampala study. Thus the current results effectively replicate those from the Kampala study that was also longitudinal and involved uncomplicated/mild malaria children. Notably, in the present study, a few individuals were found to be homozygous. While homozygosity for iNOS2 −954 G>C was not shown to be protective against malaria incidence, the actual number of such homozygous individuals was too small to empower valid comparisons, but it is noteworthy that the present results are in agreement with an earlier study in Uganda [20].

For sickle cell trait and G6PD genotypes, no significant difference in malaria incidence could be detected. However, with age stratification some differences were noted. Sickle trait carriers less than 1 year experienced higher malaria incidence than those with normal haemoglobin. This observation is in contrast with several other investigations in which the sickle cell trait was associated with protection against both severe and uncomplicated malaria [11]. The present results also fail to compare with conclusions from a study in Mali in which an age stratified analysis showed sickle trait-mediated malaria protection to be more evident in early childhood [39]. Possible explanations for the lower incidence of uncomplicated malaria among HBB wild types in the current study could relate to protection conferred by fetal hemoglobin and maternal antibodies during early childhood [40–42]. However the present study did not assess for the levels of fetal haemoglobin and maternal antibodies in infancy, thus other studies would be needed to confirm this relationship. Nonetheless, the finding of no significant difference in malaria incidence experienced by sickle cell Hb heterozygotes and normal Hb individuals when the whole children’s cohort (0.5–9 years) was analysed is consistent with another longitudinal study in Uganda in which no significant relationship between the sickle cell trait and malaria incidence was observed [20].

Similarly, only G6PD *c.202 G*>*A* homo/hemizygous children of 1–3 years showed higher malaria incidence than their wildtype counterparts (Fig. 2). On the contrary, a few studies have found an increase in the malaria incidence among female G6PD *c.202 G*>*A* heterozygotes [20, 21]. Larger studies examining the effect of G6PD deficiency on the incidence of uncomplicated malaria are needed.

Some children with the sickle cell trait also had the G6PD *c.202 G*>*A* variant. However, with only 3.9% children (16 of 414) bearing HbAS plus G6PD *c.202 G*>*A* mutation, we could not accurately assess the extent to which G6PD deficiency affects the influence of sickle cell trait on malaria incidence. Studies with larger sample size are needed to test this relationship.

## Study limitations

There were some limitations in this study. The primary outcomes for malaria as defined in this study were a combination of being unwell or ill, any level of *P. falciparum* parasitaemia plus present fever or reported history (within past 24 h) of fever. Unfortunately, other causes of fever (besides *P. falciparum* parasitaemia) such as viral infections were not adequately investigated and thus could not be ruled out. The relatively large proportion (51.3%) of children without any reported symptom of malaria throughout the follow up period of 1 year is interesting, as this finding could partly be due to acquired immunity, other unknown host factors, parasite variance or environmental factors. At any rate, some of these apparently resistant children (51.3%) could as well have experienced one or more attacks of asymptomatic *P. falciparum* infection that were not included among annual malaria episodes as defined in this study.

In addition, except for ABO blood groups, this study did not investigate other red blood cell (RBC) variants that could potentially modulate malaria incidence such as thalassaemias and other haemoglobin variants [39].

## Conclusions

This study showed that 26.6, 22.7 and 17.3% of the population in Iganga clinical trial site carry the sickle cell trait, G6PD c.202 G>A and NOS2 −954 G>C mutations, respectively. The most significant age-independent effect was the heterozygous NOS2 −954 G>C mutation, which showed reduction in incidence of acute malaria infections. Given the high frequency of sickle Hb, G6PD and NOS2 gene mutations in the study site, screening of at least sickle Hb and G6PD in regional endemic areas should be considered so as to ensure proper management of patients including those with acute hemolytic anemia.

## Abbreviations

G6PD: glucose-6-phosphate dehydrogenase
NOS2: inducible nitric oxide synthase 2
NO: nitric oxide
SNP: single nucleotide polymorphism
WHO: World Health Organization
